# Women with postpartum depression: "my husband" stories

**DOI:** 10.1186/1472-6955-8-8

**Published:** 2009-09-05

**Authors:** Phyllis Montgomery, Pat Bailey, Sheri Johnson Purdon, Susan J Snelling, Carol Kauppi

**Affiliations:** 1 School of Nursing, Laurentian University, Ramsey Lake Road, Sudbury, Ontario, Canada; 2 Canadian Mental Health Association, Sudbury Branch, 111 Elm Street, Sudbury, Ontario, Canada; 3 Sudbury & District Health Unit, 1300 Paris Street, Sudbury Ontario, Canada; 4 School of Social Work, Laurentian University, Ramsey Lake Road, Sudbury, Ontario, Canada

## Abstract

**Background:**

The research on Postpartum Depression (PPD) to date suggests that there is a knowledge gap regarding women's perception of their partners' role as carer and care activities they perform. Therefore, the purpose of this study was to describe women's understanding of their partners' or husbands' involvement in the midst of PPD.

**Methods:**

This study used interview data from a larger study of northern and rural Ontario women's stories of help-seeking for PPD. The interpretive description approach was used to illustrate the complexity of women's spousal connections in PPD. Data from a purposive community sample of 27 women who self-identified as having been diagnosed with PPD was used. From the verbatim transcribed interviews a number of data excerpts were identified and labeled as "my husband" stories. Narrative analysis was employed to examine these stories.

**Results:**

During this time of vulnerability, the husbands' physical, emotional and cognitive availability positively contributed to the women's functioning and self-appraisals as wife and mother. Their representations of their husbands' 'doing for' and/or 'being with' promoted their well-being and ultimately protected the family.

**Conclusion:**

Given that husbands are perceived to be central in mitigating women's suffering with PPD, the consistent implementation of a triad orientation, that includes woman, child and partner rather than a more traditional and convenient dyadic orientation, is warranted in comprehensive postpartum care. Finally, this study contributes a theoretical understanding of responsive as well as reactive connections between women and family members during the postpartum period.

## Background

The complex relationship between Postpartum Depression (PPD) and the marital relationship is well-documented in the nursing literature. Numerous studies have consistently associated marital factors such as conflict, dissatisfaction and support with the risk for PPD [1]. Beck [2] in a meta-analysis of 84 studies published during the 1990s, found that marital satisfaction had a moderate predictive relationship with PPD; this finding is supported by O'Hara and Swain's earlier meta-analysis [3]. Such findings emphasize that, for couples with strained relationships, the transition process associated with childbirth is that much more challenging.

During this transition, family integrity may be compromised by the presence of PPD. Maternal depression has been commonly associated with negative relational distress such as fear of being rejected by the partner, misdirected anger, withdrawal, martial dissatisfaction as well as poorer communication of needs and expectations [4,5]. Paley et al. [6] contend that during this period of adjustment it is typical for the developing partners and parent-infant relationships to become stressed. For successful relational adjustments the presence and involvement of both partners was found to be essential. Dressel and Clark [7] reported that PPD also threatens interdependence within couples by challenging their notions of family care dynamics. Further, according to Beck [8] the traditional family care role expectations required readjustments related to men's role as 'carer' for their partner.

Some authors have suggested, however, that this carer role has been overlooked by health professions; an omission partly attributed to postpartum nursing's traditional focus on the mother and infant dyad rather than on a family perspective [9-11]. Others have found that care giving is also seldom associated with the traditional ideals of masculinity [12,13]. These authors determined that women with PPD nevertheless sought partners' or relatives' assistance to lessen their suffering and to assist them with functioning. Page and Wilhelm [14] found that a woman's perception of spousal support in depression was positively related to a decrease in reported symptomatology. Their conceptualization of relationship quality, however, was imprecise.

In summary, the research to date suggests that there is a knowledge gap regarding women's perception of their partners' role as carer and their involvement in care activities [1,5,11,15]. Although there are several quantitative studies examining relationships among maternal depression, marital conflict, support and/or satisfaction [1,16], less is known about how women perceive their spousal interactions in the context of a mental disorder such as PPD [5,13,15,17]. Bost et al. [16] have reported that women's perceptions of relational experiences with husbands tend to be among the most salient predictors of their adjustment and well-being. Therefore, the purpose of this study was to describe women's understanding of their partners' or husbands' involvement in the midst of PPD. This study used interview data from a larger study of northern and rural Ontario women's stories of help-seeking for PPD.

### Literature Review

PPD is a serious, non-psychotic condition affecting approximately 13% of new mothers globally [18]. Oats et al. [19], in a descriptive qualitative study, explored PPD attitudes and beliefs of new mothers, their husbands, relatives and health care providers in 15 health centers located in 11 countries. Across all centers, participants described 'morbid unhappiness,' a condition comparable to Western's Diagnostic and Statistical Manual of Mental Disorders, 4^th ^edition, Text Revision (DSM-IV-TR) diagnosis of PPD [20]. In eight of the 11 countries, participants viewed a combination of stressed marital and family relationships, women's fatigue, and perceived lack of spousal support as being associated with the experience of maternal depression. These researchers recommended the importance of interventions aimed at strengthening the immediate family's acceptance and understanding of this universal condition. Consistent with this view, Rodrigues, Patel, Jaswal and deSouza [21] found that non-Western and Western women describe their PPD experiences through a social rather than a biomedical lens. That is, women, regardless of culture, usually interpret their postpartum emotional distress in the context of the perceived quality of their social connections. Scrandis [15] found that women with PPD described these positive connections in terms of mutual empathy and empowerment; these were dynamics that allowed them to interpret their symptoms and to cope.

There is a preponderance of evidence suggesting that positive marital relationships protect women's health in the postpartum period [14,22,23]. For example, Dennis and Ross [1] examined the relationship between 396 women's perceptions of husband support and the development of depressive symptoms. The measure to assess the dimensions of support, inclusive of appraisal/emotional, informational and instrumental, was based on literature and expert review exclusive of women with PPD. These authors suggested that women were more likely to develop depressive symptoms if they perceived that their husbands socially excluded them, discouraged them from seeking help, or did not recognize their efforts to nurture the infant at two months postpartum [1].

Further, in their examination of paternal support and maternal depression, Smith and Howard [24] claimed that the types and amount of paternal support change during a couple's first two years as new parents. They found that, at four months, nearly 57% of over 582 women reported that their husbands provided at least five types of support as compared to 45% at 24 months. Change in support was related to maternal depressive symptomatology. At 24 months, decreases in support were related to increases in depression. Decreases in support were unexpectedly associated with decreases in depression at four months. These researchers suggested that the lessening of protective processes provided by men during this early transition do not function as anticipated. This confounding result may partly be explained by Bell et al.'s [25] work with 18 new parents. They suggested that couples were often involved in particularly complex, "messy," nonlinear relational processes in the early postpartum period, a required phase of developing new ways of being together as partners and parents.

A number of researchers have attempted to understand mothers' complex subjective experiences in PPD [5,26,27]. Beck [8] conducted a meta-synthesis of 18 qualitative studies based on 309 women's experiences of PPD. She suggested that across this work the experience described by the women followed an individual trajectory influenced by biopsychological as well as social processes. Many of the included studies (61%) appeared in the nursing literature. Across the studies four themes identified by 309 women were incongruity between expectations and realities of motherhood, spiraling downward, pervasive loss and making gains. With respect to their role as wife, women were ashamed to approach their partners for help. If they disclosed their symptoms to their partners, women often characterized their partners' response as non-comforting. This, in turn, contributed to their sense of isolation and strained marital relations.

In subsequent work, Everingham, Heading and Connor [28] explored six couples' understanding of their experience of PPD. They found that all women identified a priority need to have a partner's understanding of their emotional distress. In turn, they contended that understanding lessened relational conflict as well as protected women against being labeled as 'incompetent' mothers. Although husbands recognized their spouses' need for understanding, they found that husbands often misunderstood their partners' intended messages. Expressions of distress were interpreted through a physical, personality, or psychological lens rather than through the wives' frame of being the 'good' mother. Couples' understanding of PPD from divergent frames of reference appeared to impede their adjustment during this inherently stressful transition period. Tammentie et al. [5] studied the association between women's depressed mood and family dynamics in 389 two-parent families. They found that mothers with depressive symptoms generally reported less positive family dynamics compared to their partners. Both women and their partners reported less mutuality compared to non-depressed couples. These research studies build on the work of Bost, Cox, Burchinal and Payne [16] who asserted that in the presence of PPD, husbands' and wives' perceptions of relational "give-and-take" are incongruent.

A number of researchers suggest that the emotive and social implications for husbands whose spouses are diagnosed with PPD include depression, helplessness, isolation, loss of intimacy, increased marital strain and worry [5,29-31]. In another study of men whose partners have been diagnosed with PPD, men described loss of both their spouse and their once known intimate reciprocal relationship [32]. Familiar patterns of spousal interacting and addressing problems became problematic and despairing in the context of PPD. Men's efforts 'to fix' their family situation were either not acknowledged or negatively appraised by their partner [10,33]

## Methods

### Design

The design of this study is a qualitative secondary analysis of data collected in a larger study focusing on women's help-seeking experiences. The original narrative study was a collaborative project involving women from a PPD peer support group, a public health agency and a school of nursing. The purpose was to describe help-seeking for PPD from the standpoint of the women themselves. This study's secondary analysis was a retrospective interpretation [34]; an approach involving engagement with the data beyond the self-evident - including both the assumed knowledge and what has already been established - to see *what else *might be there [35]. This was particularly relevant since data about "my husband" had captured the research team's attention in the primary study, but had not been fully examined. The *what else *in the "my husband" data set were women's perceptions of their connections with their partners relative to help-seeking. To interpret this inter-relational dynamic in women's efforts to seek help for PPD, questions such as: "What is happening here?" and "What might this mean?" [35,36] guided the researchers through this secondary.

### Setting

The study was conducted in a northeastern Ontario region with approximately 158,000 residents, 10% which are women between the ages of 20 - 34 years of age ([37]). This mid-sized urban community includes the total population of smaller rural regions that range from 1,000 - 9,000 residents. A priority regional health care need is the development of a comprehensive women's wellness program ensuring a variety of services including access to appropriate mental health support and counseling [38].

### Sample

In the primary study, purposive and snowball sampling techniques were used to recruit a community-based group of women. Verbal and written announcements about the study were distributed to a postpartum peer support group, a community mental health agency, a public health agency and family play centers. The inclusion criteria for the women were: 18 years of age or older, French or English speaking, self-identified as having received a diagnosis of PPD and had sought services for this condition. The sample for this analysis included 27 women in their early 20's to mid 30's, all of whom had sought medical services for their PPD symptoms within 12 months of their children's birth. As a result, all of the women were prescribed medication. As a family, all of the women were living with a partner and parented one to four children. The length of their spousal relationships varied from one to ten years. Sixteen (59%) of the participants identified themselves as living in communities with a population of less than 9,000 residents. All of the women spoke of being unprepared for the pervasiveness of PPD in relation to the physiological, psychological, emotional, social and existential aspects of their lives. Women described the onset of their symptoms in relation to a specific time period and their perception of their husbands' response (see Table 1, 2 and 3). Three women indicated that they had symptoms of depression prior to becoming pregnant, seven women reported the onset of symptoms during pregnancy and 17 (63%) described the appearance of symptoms following the baby's birth.

**Table 1:** Mothers reporting pre-pregnancy onset of depressive symptomatology and initial reference to husband

**Mother**	**Onset of Symptoms**	**Initial Reference to Husband**
M2	...within the first 12 hours I knew that I was not doing well/like it was just that quick	I was pretty ill so my husband could probably tell a lot more about what happened ... going home [from hospital] I expected it would get better
M15	I struggled with [undiagnosed] depression from childhood/[my child] was a planned pregnancy ... and then afterwards [after birth] there was a sudden change to where I'm speechless	I remember when we [her and partner] first got there [at emergency department] there were no beds ... so anyway we just - we're just gonna go home/[a family support person ] came and sort of smoothed things over
M26	... after my [first child] was born/I saw [name of a previous psychiatric] by the time she was six months old/the depression and anxiety panic comes a lot at night cause I wake up and just think/"what am I going to do?"	Just before [child's] first birthday my husband lost his job

**Table 2:** Mothers reporting prenatal onset of depressive symptomatology and initial reference to husband

**Mother**	**Onset of Symptoms**	**Initial Reference to Husband**
M5	... I believe that I was depressed during the pregnancy but no one identified it/six months after [my baby] was born was the critical time ...	... it was only my husband that knew what was going on. ... like it was pretty stressful kind of time for us/trying to get through it
M7	...the whole pregnancy I felt like I didn't have any control ... after coming home [from hospital] like so depressed ... I didn't feel right	I did hold them [thoughts, feelings] in and then I told my husband because he said/"you know/you're not/you know you're changing ... I don't know how to explain. ... Talk to the doctor"
M9	When I was pregnant with [my child] um I was very detached/early not attached ... it started from me when [my child] was born	... I just keep telling [name of partner] I am not ready to have a baby [following two family losses] and then unplanned we got pregnant
M11	Even before I had the baby I look back in my journal ... a month before/a month before I had the baby	[My husband] kind of backed off like I think he didn't know what was going on/he knew what was happening all along but he didn't say anything
M12	... about the second trimester of my [first] pregnancy/crying all the time um had suicidal thoughts ... it took me about nine months to finally seek help from the doctor	I was still crying and tearful and moody ... like we [my husband and I] kind of noticing the signs now/obviously
M18	My seventh month of being pregnant I noticed that I wasn't excited/I was really nervous and fearing everything .../so anyway/I had a good delivery and even the same day/the same evening I didn't want to see [my child] ... when I came home [from hospital] I'm thinking my world just fell apart	... my husband stayed with me while I was in the hospital/so I kinda let him help out/we do almost everything ... [when child was about one month of age] I finally broke down and told my husband there was something wrong because he when back to work and I couldn't be alone because I feared everything
M22	I first became aware of depression when I was six months pregnant	I remember looking at my baby who was crying and my husband was trying to comfort her/I had my first panic attack ... my husband went for help

**Table 3:** Mothers reporting postpartum onset of depressive symptomatology and initial reference to husband

**Mother**	**Onset of Symptoms**	**Initial Reference to Husband**
M1	... around 4 1/2 months and ah I started to recognize that there was something terribly wrong	I told [my husband] I hadn't slept in two weeks ... and my husband ... he drove me to the hospital
M3	... the first month ...the mood ... sit there by myself and cry and then feel guilty ... I suffered from a lot of anxiety	... my husband he was pretty much caring for [the baby] through the night/I would care for [the baby] during the day and as soon as he would come home I was out of the picture
M4	... I knew I needed help at three month and I waited for the four month checkup with the baby	... make everything look perfect on the outside ... [the doctor] asked me how much I was sleeping ... my husband said/"What!"/he had no clue that I wasn't sleeping
M6	I was getting the anxiety that you know ... but I know one night I was really bad like ... close to four months [postpartum]	Then one night I thought I heard her cry all night/my husband said she didn't cry at all and so I guess my nerves were really bad and then one day I said like to my husband/"I couldn't/I just couldn't deal"
M8	... [my child] was 13 months and um started having real anxiety about being alone with [my child] and um feeling very overwhelmed with her care	... my husband was out of town overnight so I knew he wasn't going to be at the house that night and I was just going to be on my own and I was just very overwhelmed
M10	It started pretty much when [my child] was born and it didn't take long for me to know that it was bad	I tried to tell my husband but just like he kinda tried to deny it
M13	I had depression right of the bat like within the first month/I didn't realize when it was happening/I just knew that I was stressed out and I I felt different	My husband involved my mother
M14	2 1/2 days after I had the baby/that evening I had a panic attach/I didn't know what it was	... next day [my baby] had an appointment with the doctor/my husband and I explained what happened ... that night it happened again but much worse/my husband had to call someone over
M16	I had a bit of postpartum with my first ... with my second and in fact sought help from a doctor then/I knew right away what if was with the third one	My husband could see it in me and you know he would ask me you know/"what's going on with you and the more supportive he got the more I wanted to cry"
M17	When the baby was eight weeks/exactly eight weeks/I was having a lot of physical symptoms	... so the realization [concerning PPD] came just mainly by myself and just talking to my husband
M19	When the baby was born I just didn't feel right/I didn't feel myself	... my husband had to take me to the doctor's because I knew something wasn't right
M20	... so much pressure seemed to be on me [after my second]. I got to a breaking point where I just I can't do this anymore	I know my husband before me came to the conclusion/was telling me/"there's something wrong/there's something wrong"/but I was like/"no"
M21	...during the first year [of child's life]/I didn't know it was depression ...I thought I was crazy/I thought there's something wrong with me mentally	I didn't show it [depression] in from of him .../he thought it was just the baby blues ... he/he did notice that I was/there was something wrong with me ... like he didn't have anything like that in his family
M23	... I was so sick right after she was born. I was so depressed	... it came to a point that I said to my husband,/"I have to be serious"/I told him/"I really get depressed and I have fear with it"
M24	Throughout the pregnancy I had never felt better in my whole life/one week after the baby was born I was sinking/I was terrified to be left alone with [the baby]	I told my husband/"don't leave me alone/I'm afraid"/his thinking was/"you are just afraid/you got to get use to [the baby]"
M25	... first two weeks of the birth of each of my two children I had moods/couldn't sleep/tired no matter what and bad nerves/I was hospitalized the second time	... after I came home from hospital [psychiatric hospitalization] I was so so anxious my husband had to take care of me and the kids
M27	... for one year after [first child's name] was born I felt like I was going crazy ... I totally changed ... I didn't feel like I was me anymore.	... my my husband thought it was post/we both thought it was postpartum depression/thought it was emotion

### Data Collection

Following ethical approval from two respective boards, Laurentian University as well as Sudbury & District Health Unit, and under conditions of informed consent, audio taped interviews were conducted by a mental health nurse (PM) and a PPD peer support worker (SJP). In the primary study, an unstructured interview characterized as a guided conversation was followed. This type of non-threatening, supportive exchange allowed women to share help-seeking information grounded by contextual details that made sense to them [39-41]. The topic of questions used to begin or guide the conversation were about their help-seeking decisions, their understanding of their support needs and their appraisal of available support resources. Each woman completed at least one audio taped interview, ranging in length from 30 to 90 minutes. The interviews were conducted in homes, community clinics or coffee shops. Four women requested a second interview to permit them more time to tell their stories. Since one of the interviewers may not have been a stranger to prospective participants, specific demographic data was not collected respecting participants' right to confidentiality. Women, however, shared information such as age, mental health histories, onset of PPD, and/or their and their partners' employment in formulating their help-seeking stories.

### Narrative Analysis

From the verbatim transcribed interviews a number of data excerpts were identified and labeled as "my husband" stories. The label of "my husband" was the most common term of reference used by women when they spoke about their partner. The broad underlying premise of narrative inquiry within social science research is the belief that individuals most effectively make sense of their world and communicate these meanings by (re)constructing stories or narrating them [40,42-48]. Therefore, it is important to examine these structures within interview data. An eclectic narrative analysis strategy incorporating Labov and Waletzky's [49] functional analysis model of individual stories and Agar and Hobbs' [50] strategy of identifying story coherence across interviews developed by the second author (PB) was used to interpret the identified stories [51-54]. This analytical approach was used because it provides an explicit analysis process and facilitates the interpretation of meaning in complex stories [55].

In the primary study, "husband" stories were identified as first-person event-specific and generic stories but not systematically explored by the research team. For this study, using unmarked transcripts, the first two authors individually deconstructed each "husband" story into the following story elements: abstract, orientation, complicating action, resolution, evaluation and coda, as defined by Labov and Waletzky [49]. These authors then jointly reviewed and compared these elements within and across the stories to assess shared content and structure. In addition, they examined the stories for the inclusion of discursive and rhetorical devices [56]. For example, participants frequently repeated words or phrases and used direct quotations to enhance the credibility of their accounts. Finally, these authors explicated an interpretation of the story meanings (content themes) and function (utility) of these meanings to develop an understanding of husband behavior in the midst of PPD for the participants.

To demonstrate meaning and function of the husbands within the context of PPD, as described by these participants, the results are presented in two sections: the stories and a case example. Within the stories section, story structure and content as well as story meanings and function are described. Next, a case example is presented to illustrate one participant's understanding of her husband's involvement in the midst of overwhelming symptoms. The case example, presented using Labov and Waletzky's [49,57] structural elements, is intended to make the analysis process transparent [48,58]. These presentation strategies facilitate the reader's ability to examine the analytic logic and researchers' interpretation process [35,59].

## Results

Each of the 27 participants shared information about their "husbands," his role as an adult partner rather than that of father during PPD. In total, the women contributed 153 stories about their partners' availability. Using explanatory narratives [55] to communicate complex points of view that might be otherwise hidden to the outsider, the women characterized two types of husband availability: 'doing for' and 'being with.' These accounts illustrate their insider perceptions about their husbands' presence, both tangible and intangible. The data in each section is presented in an un-tidied format to preserve the integrity of the women's account.

### Structure and Content

Across the interviews the women talked about their perceptions of their husbands' availability. Although their descriptions of husbands' availability differed, all stories were bound by the common theme of PPD symptoms. Their stories portrayed two kinds of husband availability: 'doing for' and/or 'being with.'

### Their PPD Reality

The women's symptomatology experiences framed their stories about their husbands. As shown in Table 1, 2 and 3, the onset of their symptoms was associated with a specific timeframe. A number of women explicitly described their symptoms as "overwhelming," "unpredictable" and/or "incapacitating" in terms of the impact they had on their ability to meet their expectations of mothering:

M14: *That night, it [the panic attack] happened again, but much worse .../I couldn't even talk on the phone .../cuz I didn't have the energy .../and all of a sudden I just couldn't calm down/and I was having a hard time breathing/um/my heart was racing/I got my husband/I told him "I think I'm dying"*

M15: ... *I didn't recognize depression. I grew up in a family that believed/pull up your socks and um/...I felt very isolated and really unhappy and then guilty about it. I remember that I was really worried, very worried/I had what turned out to be anxiety obsessional images of [child] that weren't so but I was so unstable and that happened so quickly*

M19: *And it just worsened and 3 days after I was home ... I didn't want anything to do with him [the baby]/and he cried and cried, he had colic ... I was on medication 3 days after the baby was born*

M27: *For that year [first year of her child's life] it was like I just wanted to be away from everybody and that's not like me ... I just wanted to sleep and sleep and sleep/I was not the same person ... I noticed the change in myself, my personality and my character/I just I didn't feel like I was/it felt like somebody took my body*

M2: *Like I can't feed [the child] properly, I can't change her properly/like I touch her she cries/I put her down she cries/like just somebody has to take care of [the child] because I can't ... I was getting pretty frustrated with everything*

M20: ...*no matter how prepared you try to be you are never prepared enough/it is something that just happens and people get through it and that's it taken for granted ... your world is just so distorted and I think that people around you are kinda going/"Oh my God" and they really don't know what to do either*

M21: *I wasn't myself/I wasn't acting like myself/I wasn't doing things I usually did/like it was all of a sudden/I didn't care anymore*

Women often spoke about their difficulties in verbally articulating their suffering so that their husbands understood their needs. As M3 explained, "I don't even know how to explain it/it is hard to bring up the words." They acknowledged that difficulties in expressing themselves clearly contributed to family chaos and strained marital relations.

M9: ... *and he wasn't hearing me/wouldn't listen to what I was saying/and he was walking away from me/and I grabbed it [a toy] and whack/so hard on my head that it split my head open/and blood starts coming down*

M1: ... *my husband was like "what the hell is wrong with you?" I said "you [my husband] have to call [someone for help]"... my husband was blind he really was/he was really blind to everything that was going on/and he was not a really big help in the beginning/I sort of felt like I was doing everything on my own you know*

M14: ...*I was thinking why is my husband planning for [the child's] future as an adult/and I was just constantly thinking that she was going to die because I was neglectful/or because I was doing something/that I ah/I in some ways/I thought/because I couldn't breastfeed I wasn't a good mother*

M27: ... *even my husband found it very hard to cause he's always tries to keep the peace like so it was very difficult for him to not upset me. It's just/I wasn't the same person*

M1: *So my husband ... came out and said "well you know you're kind of not yourself"/and I was like "get lost"/... this was mid August/by September he was like/"no you need to go and see someone"*

M3: ... *it's always that my husband/it's always/he said the same thing all the time/"I can't read your mind"*

In addition, some women did not initially interpret their experiences of fatigue, irritability, discomfort and/or tearfulness as PPD. Through their husbands' direct sharing of their observations, these women began to question if their inner psychological experiences were becoming apparent despite their efforts "to hide" symptoms or "not to tell" their husbands. Later, husbands confided that they sought validation of their genuine concern that "something was not quite right." Often they initiated consultations with immediate female family members. The husbands' worries were rooted in their knowledge of wife as a person.

M7: ... *I told my husband about it [PPD] because he said/"you know/you're not/you know you're changing/you've changed [a lot]"*

M13: ... *My husband involved my mother .../and actually he/he talked to my mother and said/"there's something wrong with her/this is not normal"/and my mother took him seriously and I really appreciated that cause ap-apparently he said I was at like three in the morning*

M20: *It is almost/are/there is a time when a mother is ready to hear/we need help ... my husband telling me "there's something wrong/there is something wrong" but I'm like "no its your family, it's you" ... there seems to be a certain time when the partner can say to you there's something wrong and you listen*

M3: *He [my husband] did notice that I was/there was something wrong with me but he couldn't see what it was/he didn't know about depression that much either/like he didn't have anything like that in his family so *...

M4: *My husband/he said there's something wrong/you need help. ... so he did question but he himself didn't know where to go or what to do either*

### Husbands' Availability

The husbands' availability was of two types: 'doing for' and 'being with,' with the latter being the more typical mode. His physical presence, regardless, lessened their fear of "going crazy." Being "alone" in the home was perceived as begrudgingly tolerable, especially when husbands were contract or shift workers, requiring them to be out of the home for extended or unpredictable periods of time.

M8: *I remember really well/I was picking [the baby] up from daycare at the end of the day and my husband I think was out of town overnight/so I mean I knew he wasn't going to be at the house that night/and I was just going to be on my own/and I was just...very overwhelmed*

M14: *The first few weeks when my husband went back to work I was afraid to be alone at night because that's when it [symptoms] got the worst*

M18: ... *there was something wrong/[my husband] went back to work and I couldn't be alone because I feared everything/so either I spent my days at my parents or my mom came for the day until [my husband] came back*

M1: *My husband worked the graveyard so I was alone all night with [the baby] and/and then he was sleeping during the day so I was alone with [the baby] all day*

M22: *My husband returned to working long hours/I was all alone/I never felt so helpless*

M3: *My husband did/he's the one that called the health unit/he's the one that found about the group/...before I used to be the one to call if there's anything*

M24: *It was just don't leave me alone because I was afraid ... like I got overwhelming fear that I had [husband's name] go to the emergency with me and we talked to the doctor *...

Additionally, their husband's physical presence was represented as an opportunity for women to verbally express their worries and uncertainties. Overall, the women were strategic about what, how and when they talked to their husbands. A criterion they used to judge the nature of their disclosure was their assessment of the potential risk or benefit. Women did not want to risk "further isolation," "frightening" their husbands or having their partners distance themselves by "never talking about what happened" in illness.

M6: *I like/I was really pretty down at that time ... I was really down and I wanted to talk to a friend who used to work in [social services]/I wanted to see her/the next day I get a call/he [caller from social service] said that the staff mentioned that I was pretty down and I wanted to go see Children's Aid/and that I should go/and then I told this to my husband/he's all worried about Children's Aid coming over/I said "no no"/I said I said/"I was having a bad day"/I guess that's why [my husband] freaked*

M14: *It's horrible/and so like I I had dreams that I was trying to do stuff to myself and my children/but I knew/and I brought it up to the doctor/he [the doctor] said/"if you have bad dreams/you need to get them out/whether it's to your husband"/or I didn't want to scare my husband more than I already had [laughter]*

M3: *I told my husband/I told him like I would dream of falling down the stairs/so I break my leg or so I break my neck or something/it wouldn't be me doing it on purpose ...why can't I just fall and die or something like/but/how can I even think that way? I couldn't/I know its illness*

The above three examples about "telling my husband," however, did not include a description of husbands' apparent responses. Rather, within the context of their spousal relationship, the women implied that their verbal utterance of events or thoughts were "heard," thereby, reaffirming their interpretation. This partly may be associated with women's perceptions that their husbands' "didn't know what to say/think or do." The following excerpts illustrate how these women connected with their husbands particularly when their mothering competency was questioned by others.

M6: *I would go to the mall often/I just needed to be out and ... [my friend] said to me "well your baby [at 3 months old] is lonely too/she needs more than to be a mall baby"/and that really bothered me/because I said to my husband "how did/can [my friend] judge me like that?" "How did [my friend] know my baby was lonely?" .../he [my husband] just/he just *...

M17: */um/and at that point I realized okay you know/it's not something physical/it has to be emotional/so the realization came just mainly by myself and just telling my husband*

### 'Doing For'

'Doing for' stories involved the husbands' response to the immediate needs of the family, both spouse and child. Circumstances compounded by PPD placed husbands in the challenging position of simultaneously being both family provider and carer.

M13: ... *my husband couldn't get the/more time/well he was offered more time off work but there was no money there and we need to bring a pay check in pay for the house/pay for the house/all these other things*

M17: ... *bringing in take-out once in a while or/you know/just taking the baby out so I can have some alone time/um/picking her up from the babysitters so that helps to/so a lot of just little things that amount to a lot*

M5: ... *my husband with the baby at home and he's saying this isn't going away/you need help you know/so he when in to talk to [his physician]/[the physician] told him that I have to come in/he took me in the next day and that's when the referral started happening/... it really helped with [partner's name] being able to change the diaper/make bottles*

M20: *lots of time I would tell him to do certain things and he'd do it*

M26: *he was supportive by taking any kind of job because of our financial stress*

Frequently, in relation to their perceptions of "losing control," women explained how their husbands undertook care tasks to mediate symptoms. For example, they called substitute care providers, arranged child care, administered medications, or made themselves available to transport the women to healthcare providers.

M3: ... *I couldn't ask for help for babysitting/for anything/he [my husband] had to/be- because to me if somebody says "no I can't tonight"/then it was like/it's rejection*

M13: ... *my husband pretty much had to force the pills down my mouth/cause I was just like/"I'm not taking this I'm not that that I don't need this" and he'd like say "take your pill" and I would hate him for it*

M14: *um/I had to call a friend/my husband had to call a friend over/a friend I know that works for the [a health care agency]/and she came over while my husband could attend to the baby/and my other daughter/um/my husband or her had to help me nurse the baby*

M16: ... *husband encouraged me actually to call [a community support service] when I told him about it/he said/"well why don't you get out/get out of the house and call"/and yeah well I'm dealing with it and he's like/"no go and do this"/so he encouraged me*

### 'Being With'

'Being with,' stories involved the husbands' affective presence that also promoted the women's sense of security. 'Being with' their husbands afforded women the opportunity to discuss their worries without fear of alienation. They perceived their husbands as respectfully close. Some women described their husbands' repeated offers to mutually share the day-to-day responsibilities as husband and wife.

M17: *[my husband's] been wanting to talk about it like asking me how I'm doing/and you know/"what are you thinking about"/and he's very tuned into my feelings so sometimes he'll know "okay what's going on?" "what are you thinking about?"*

M18: *I finally broke down and told my husband there was something wrong/so I kinda let him help/we did almost everything because I feared everything/my husband/um a lot of his care/I was able to talk with my husband mostly*

M25: *I don't get along with my family/my husband would run interference by telling my mother it wasn't a good time to come over/making sure people who upset me stayed away*

M20: ... *there seems to be a certain time when the husband can say to you/I'm frustrated and I don't know what to do*

M4: ...*like [name of partner] being there and him learning a lot about the stuff that was happening*

Aware of the impact of their symptoms, women gave their husbands permission to be their co-voice with trusted others such as familiar healthcare providers. In these cases, 'being with' involved his responsibility of interpreting or decoding the situation for their wives or families following a joint discussion.

M8: *I made my husband read one of those checklists about your feelings/and I said "I'm not gonna know if this happens to me/so you've got to look for these things"*

M14: *And um, the next day my son had an appointment with the doctor/my family doctor/so when I went/um my husband and I explained what happened...one of my relatives said um to my husband/"oh/I seen a commercial about this postpartum depression" and "oh/[my husband] you know what/she doesn't catch that" and he is like "she does have it/you don't catch it"/you know/"it's um/it's hormonal/it's not about not managing having two kids"*

M1: ... *[my husband] would let me know that I was getting better and he just tell me/"it's ok"*

M2 ...*like my husband and my relationship is way better although I feel guilt on a lot of fronts/I mean we've we've come from so far*

M3: *We'd had to figure out a plan of attack we are going to take*

M10: *We were in such a strange place/we wanted our privacy ... we called [a community service] because we didn't know where to start*

Husbands were presented as responsible for preserving their wives' as mothers with external family members and, in limited cases, with a close family friend. Again, the public image 'as mother' was often a product of joint discussions bound by the family's privacy and cultural traditions.

M13: *I had my husband and my mother .../he'd say [to my mother]/"she's pacing"/and she's like/my husband brought my mother just so that he would have the nerves to tell the doctor what he was seeing with me*

M1: *I told my husband to call my good friend ...my husband called her in the middle of the night and told her what was going on/and she said "you gotta take her to the hospital"*

M19: ... *my husband and I explained to her [health care provider] what was going on and she's the one that said/"yeah/probably postpartum depression" ...um restarting my relationship again with my husband*

### Meaning and Function

The meaning statements within stories address the question, "Why are the stories told?" The meaning of the availability stories was that partner as husband (and to a lesser extent father) was needed by the women who were struggling with motherhood in the context of PPD. The inclusion of messages such as "what can I [as husband] do to help?" "I told him," "I was heard," reflected women's perceptions of their husbands' tangible and intangible central involvement. During the period of adjustment following childbirth, 'doing for' and 'being with' stories revealed types of husbands' availability - physically, to do; affectively, to comfort, and cognitively, to interpret - each of these modes presented as integral to their integrity as women and shaped by the context of mothering.

Familiar patterns of availability seemed compromised during this stressful transition and compounded by the presence of PPD. The women's stories illustrate perceptions of the intrinsic care provided by husbands. Husbands' engagement in different modes of availability and their connectedness with their wives as trusted other seemed particularly adaptive. Variability in availability seemed to be valued given the mothers' description of the ambiguity, unpredictability and unexpectedness of PPD. In retrospect, husbands communicated their availability through expressions such as "its okay," or through actions such as taking the day off work, or even when he was directive in stating, "take your pill." Participants evaluated the husbands' availability as purposefully caring for them as women.

### Case Example

Nancy (M11) was a young first-time mother, on maternity leave from her professional occupation. She lived with her husband and infant in a residential neighborhood. Her husband's employment situation was tenuous given that was a contract position in the service industry. Her own mother did not live in the region and was not accessible for support after the birth of Nancy's child due to commitments within the extended family. Her mother-in-law resided in the area and was able to help if asked. She described the postpartum period as a time when "my life was extremely scary." Her story included the emotional, physical, social and functioning implications typically associated with PPD. Nancy's desire was "to be the best mom that was possible." Thereby, she recounts her preference to be independently responsible for all the child's nurturing, so that he "could sleep" and continue to fulfill his obligations as provider for the family. Within two months, however, Nancy realized that she was "completely lost" and knew, "in [her] heart" that she "needed" her husband to become more involved so that she could attend to her increasing mental health needs. The following story illustrates an aspect of Nancy's husband's availability. It is presented using Labov and Waletzky's [49,57] structural elements to make the analysis process transparent. Each line of the dialogue is numbered and represents her interaction with the researcher including speech interruptions, false starts, and overlaps in speaking turns. This story was prompted by the researcher's inquiry and in turn, Nancy's response without further dialogue from the researcher.

Story stimulus:

533 Researcher: *To clarify, a community nurse came to visit?*

Orientation:

534 M11: *I had a visit from a nurse/and that was it/I had a visit*

535 */someone came in and weighed the baby/they weighed the baby/she*

536 *did this to see if the baby was drinking?*

Complicating action:

537 *She [nurse] asked me "are you breastfeeding?"*

Evaluation/Complicating action

538*"Is any milk coming in?"/she kept focusing on the breastfeeding part *539*you know/at this time like/put on your smiley face*

Complicating action:

540 *umm I wanted to tell her "listen/it so hard ..."*

Resolution:

541 *I told my husband after that*

Evaluation:

542 */I can remember how this visit made me feel bad/it made me guilty*

Nancy's reference to her husband after experiencing an unsettling mothering event seems tangential because she does not verbalize his immediate response. Her chosen disclosure of this personal experience to him as husband infers her perception of his availability as a safe and protective other when she herself has doubts about her adequacy of mother. Nancy's transcript continues uninterrupted with the immediate introduction of another story that emphasizes her husband's availability.

Orientation

543 cause *when my mom was here I was upstairs pumping for two*

544 *hours to get two ounces*

Complicating action:

545*/the nurse reminded me, "it's not that hard/come on I'll help"/the*

546 *baby is screaming like blue murder*

Evaluation:

547*/and the nurse goes, "it is not working"/I wanted to scream*

Orientation/complicating action:

548 *I'd remember when my mother-in-law come to visit/running up stairs *549 *and pump pump pump pump pump cause [child's name] needs a*

550 *feeding/this is what is best for the baby*

Abstract:

551 *I'm a poor mother because I can't do this/I wasn't able to breastfeed*

Evaluation/complicating action/evaluation:

552*/I bought formula but/*

553 *whenever you're in that state of mind/I couldn't/it was so painful*

554 */cry, cry cry because not even a drop was coming out*

555 *and my mom said/"listen your kid is not gonna die"*

556*/my husband said he was raised on carnation milk/honey and water 557/and he's like normal functioning*

Nancy expended much time, energy, and pain to fulfill her expected role as the "best" mother including breastfeeding. Despite all her intended efforts to breastfeed, and solicited consultations with others (nurse, mother, and mother-in-law), it was her husband's verbal response and affective presence that reassured her and lessened her guilt as a mother. In this case study, Nancy's husband was needed as she struggled to meet her ideals as a mother that were reinforced and advocated by others. Nancy's matter-of-fact recounting of her husband's seemingly purposeful and timely availability was interpreted as caring for her.

## Discussion

The initial focus on maternal help-seeking practices in PPD in the larger study generated this study's data subset suggesting the essential availability of husbands for women experiencing PPD. As evidenced in their stories, for this group of women, the availability or presence of "husband" was largely perceived as providing affirmation and security. Consistent with other research [8], these women's experience in PPD was destabilizing, and negatively interfered with their ability and desire to attend to their roles as wife and mother. To establish balance, the women required their husbands' physical, affective and cognitive involvement as they struggled with PPD. The interpretation of the husband's availability ranged from conflicting, reassuring and/or nurturing in relation to the woman's perceived health status and efforts to mother.

Although men generally desire to be supportive to their partners, the demands of infant care, even in the absence of PPD, have been found to contribute to relational adjustments and strain [60]. In the postpartum period, Blum [61] proposed that the availability of a sympathetic other counterbalances another's inherent dependency needs, whether verbalized or not. Women's risk for maternal distress increased when they find no one available to assist them in meeting such rudimentary psychodynamic needs. Basic attentive listening to a mother's concerns may address her needs without requiring her "to acknowledge them any more than [she] can." [61] The current study's findings suggest that over the course of PPD a husband's availability may need to modulate from a physical presence to a reciprocal exchange. Such unpredictable and fragile transitions appeared to communicate commitment to, responsibility for and preservation of the woman and the family. Although not explored in this study, previous research suggests that men experience their own struggles with maternal PPD while intending to lessen the suffering of their partners through attempts at involvement [4,5,32,33].

For these women, understandings of their husbands' physical availability may have been as subtle as their "sitting in the chair" or "just being in the house." Potentially, husband's physical proximity permitted a connection, even an opportunity for him to observe and protect his wife. Consistent with the findings of other studies [62], several of this study's participants did not initially view themselves as ill. Some women in this study engaged in self-silencing, an interpersonal style intended to minimize their partners' tension, worry or rejection. As a result, his availability and intentionally cautious watching because of unfamiliar circumstances may be considered a positive source of support and validation [63,64]. In addition, husbands' verbalization of their perceptions about their wives' behaviors and challenges in attending to the needs of their infants was critical in legitimizing the women's distress and assisting in seeking help.

This study's group of women reflected on the importance of 'doing for,' or instrumental support as identified in other studies [1,24,62]. Because of the women's vulnerability, 'doing for' appeared to be a one-way transaction aimed at performing a task such as driving, cleaning or administering medication. Consistent with other studies [4], emphasis was on what the husband did to "fix" emergent problems; helping to mobilize resources to meet his partner's or family's needs. For the dependence/interdependence health of the couple, our findings reinforce the importance of a network of resources to moderate the health risks for each partner. This dynamic is reinforced in Goodman's [11] integrative review of PPD in fathers; depression in one partner influences the health of the other.

Barclay and Lupton [33] found that, for men, "being there" refers to a physical and emotional sensitivity for the sake of family stability and mutual support. Although many of the men in their study remained on the "fringes of parenthood," for the first sixth months if their child's life, they were committed to providing responsive care for their partners' and infants' needs. As Goodman suggests [60], despite fathers' intentions to "be there," the partners' redefinition of roles is characterized as a process of "trial and error." From the perspectives of women in the present study, husbands' availability, as part of the transition, afforded the mothers the opportunity to verbalize select feelings and thoughts, thereby possibly lessening inner emotional chaos.

For this group of women, 'being with' their husbands was expressed in less tangible terms than their husbands' 'doing for' pattern of availability. These narratives do suggest that their spousal relationship provided the context for reciprocity as the husband listened to, advocated for, and negotiated with his partner. As Doucet [12] proposed, "being with" is an inter-subjective connection: partners interacting, learning to move through spaces such as parenthood congruent with public expectations concurrently making judgments about maintaining or avoiding social encounters. In terms of the buffering effects of spousal support in PPD, women who perceive their partners as emotionally supportive reported less avoidance behavior, greater martial satisfaction and less depressive symptomatology [1,16,65]. The combination of these 'doing for' and 'being with' results support Ontario, Canada's recent family development services directed at both adults sharing responsibility for nurturing intra-familial environment during transitions.

With regard to secondary prevention, research has demonstrated that postpartum programs emphasizing relational interventions were associated with positive maternal and family outcomes [18]. Paris and Dubus [17] explored volunteer, paraprofessional and home visitors' relational interventions with postpartum families. Home visitors perceived that their provision of instructional and emotional support, validation and affirmation positively influenced the development of partner and parent-child relationships. Although the current study findings do not advocate for care within the marital relationship to become professionalized, women recommended the need for information and skills that focus on cultivating mutually empathic relationships. Future longitudinal research should explore men's perceptions of their informal care giving processes in PPD.

The researchers of this study have not attempted to make claims of causation or truth [58,66]. Instead, through repeated engagement with the data an understanding from women's representations of their husbands' central involvement in PPD has been constructed. Several of the women who self-identified for this study had previously shared their PPD experiences through informal peer support groups, potentially influencing the results. These women may have processed a more thoughtful view of their husbands as a result of the positive effect of their support-seeking behaviors. As previously indicated, to enhance the study's trustworthiness a research team member who was a PPD peer support worker and, therefore, contextually aware, assisted the academic researchers to "see what [they could] not yet see" [35]. Further, to address representative credibility [33] in relation to women's help seeking in PPD while ensuring their confidentiality, the lack of a standardized collection of particular identifying socio-demographic information from women living in smaller communities may limit the transferability of the findings. As Thorne [33] suggests, however, we were aware that our chosen methods had to reflect rather than define our research purpose.

## Conclusion

This study described a sample of 27 women who self-identified as having PPD and their understanding of their husbands' availability. During this time of vulnerability, their husbands' physical, emotional and cognitive availability positively contributed to their functioning and self-appraisals as wife and mother. Their representations of their husbands' 'doing for' and/or 'being with' promoted their well-being and ultimately protected the family. Given that husband is perceived as central in mitigating women's suffering with PPD, the consistent implementation of a triad orientation that includes wife, partner and child as opposed to a more traditional and convenient dyadic orientation is warranted in comprehensive postpartum care. Finally, this study contributes a theoretical understanding of responsive as well as reactive connections between a couple during the postpartum period.

## Abbreviations

(DSM-IV-TR): Diagnostic and Statistical Manual of Mental Disorders, 4^th ^Edition, Text Revision; (PPD): Postpartum depression.

## Competing interests

The authors declare that they have no competing interests.

## Authors' contributions

All authors contributed to the research's conception and design. The manuscript was written by PM and PB. The other authors read and approved the final manuscript.

## Pre-publication history

The pre-publication history for this paper can be accessed here:


